# Paleoarchean plate motion: Not so fast

**DOI:** 10.1073/pnas.2218383120

**Published:** 2022-12-27

**Authors:** Ross N. Mitchell, Xianqing Jing

**Affiliations:** ^a^State Key Laboratory of Lithospheric Evolution, Institute of Geology and Geophysics, Chinese Academy of Sciences, Beijing 100029, China; ^b^College of Earth and Planetary Sciences, University of Chinese Academy of Sciences, Beijing 100049, China; ^c^College of Resources, Environment and Tourism, Capital Normal University, Beijing 100048, China

Early plate tectonics is hotly debated, and paleomagnetic data can provide critical tests (1). Brenner et al. (2) present data from Pilbara Craton from ca. 3,250 Mya indicating polar motion at speeds of ≥0.55° My^−1^. Paleomagnetism measures apparent polar wander (APW), continental motion relative to the magnetic pole, where APW = plate motion + true polar wander (TPW). (Net lithospheric motion is comparatively small.) Although the authors rule in favor of plate motion, by their own admission: “That said, it is technically possible to ascribe all observed motions to two successive TPW events.” If the APW is instead due to TPW, then the Paleoarchean data have no bearing on ancient tectonics.

**Fig. 1.**
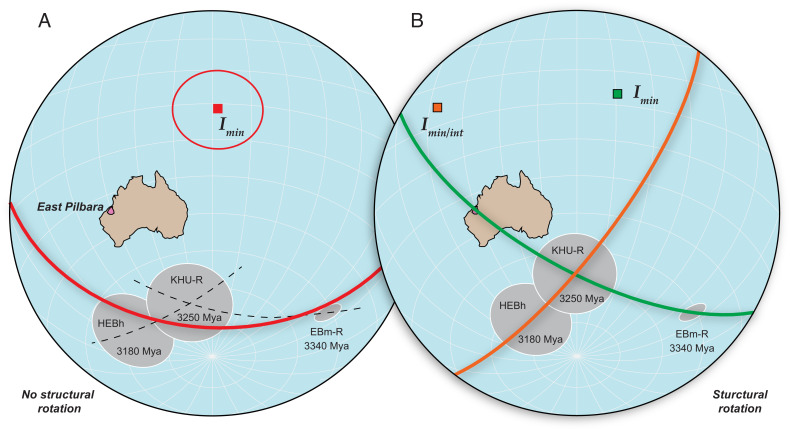
Paleoarchean paleomagnetic data consistent with a TPW interpretation. Poles without (*A*) and with (*B*) structural rotation (2). Both options are consistent with TPW, and neither necessitates plate motion. *I_min_* or *I*_*int*_, minimum and intermediate moment of inertia.

Their argument against TPW is that the two APW segments identified have orientations that differ by 48° to 90°, and “due to persistence in the geoid shape,” this is unlikely for TPW. Much of this difference derives from the structural rotation of the younger two poles from the same syncline relative to the rest of Pilbara. But the authors consider scenarios between 0 and 70° rotation because its magnitude is uncertain and may be zero. Without structural rotation, then the three poles exhibit an essentially continuous direction of polar motion, thus invalidating the empirical basis of their argument against TPW (Fig. 1*A*).

Assuming their preferred structural rotation of the young poles is correct, the two APW events are nearly orthogonal (Fig. 1*B*). A significant shift in APW orientation is entirely consistent with multiple TPW scenarios, negating also the theoretical basis of their argument. A 90° shift in TPW axes can occur when TPW occurs about either the minimum or intermediate principle axes of inertia, which are orthogonal, or during multiple examples of “orthoversion” when successive TPW intervals are shifted ~90° in longitude due to mantle convection reorganization (3). Even for smaller shifts, their presumption of a persistent geoid shape is predicated on the existence of long-wavelength mantle convection, which may not exist in the Archean (4, 5). Thus, the argument against TPW has either one of two problems: Either it is based on uncertain APW orientations or the observations are fully compatible with TPW.

**Fig. 2.**
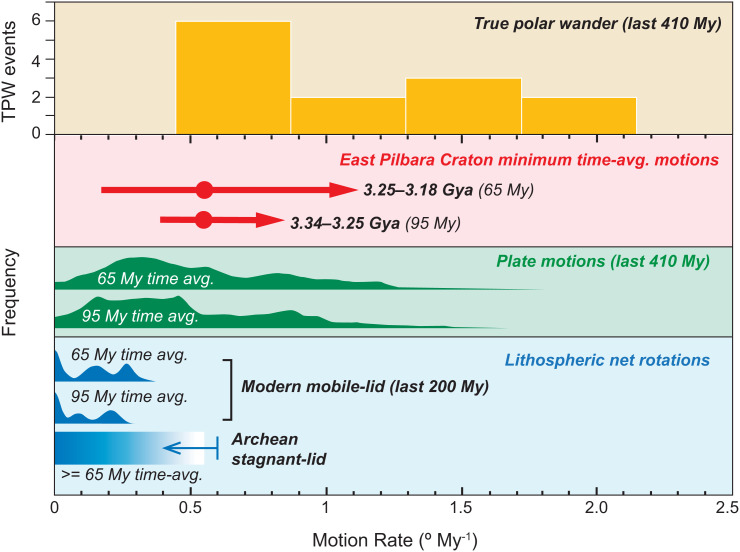
Comparison of Paleoarchean data and various forms of continental motion. The best match for the Paleoarchean minimum rates and their potentially faster ranges is with TPW (orange). All data are from ref. 2 except TPW rates from ref. 10, where one outlier plots off-scale. Plate motion and TPW rates are over the same interval for direct comparison.

APW speed is used to bolster their tectonic interpretation (Fig. 2). The Paleoarchean rates are claimed to overlap with those of tectonics, albeit only with the low-probability faster speeds. There are arguments and evidence that tectonics might have been slower in the past (6). Meanwhile, the Paleoarchean rates and ranges align more closely with the mode and median of TPW. It is unknown whether ancient TPW was faster or slower. A hotter, less viscous mantle could deform quicker, but TPW is dominantly excited by subduction (7) and Paleoarchean slabs were likely shallow (8) and/or subduction may have been globally limited at that age (9). Such a trade-off in the TPW rate over time may explain the match between the ancient data and recent TPW. With no basis for ruling out TPW, the implications of these data for ancient tectonics are rendered ambiguous or irrelevant.
